# Safety and Efficacy of Minimal- versus Zero-fluoroscopy Radiofrequency Catheter Ablation for Atrial Fibrillation: A Multicenter, Prospective Study

**DOI:** 10.19102/icrm.2020.111105

**Published:** 2020-11-15

**Authors:** Paul C. Zei, Kenneth K. Quadros, Paul Clopton, Amit Thosani, John Ferguson, Chad Brodt, Geraldine O’Riordan, Mattheus Ramsis, Raman Mitra, Tina Baykaner

**Affiliations:** ^1^Brigham and Women’s Hospital, Boston, MA, USA; ^2^Stanford University, Palo Alto, CA, USA; ^3^Allegheny Health Network, Pittsburgh, PA, USA; ^4^Beacon Health System, South Bend, IN, USA; ^5^University of Virginia, Charlottesville, VA, USA

**Keywords:** Atrial fibrillation, catheter ablation, minimal fluoroscopy, zero fluoroscopy

## Abstract

Radiofrequency catheter ablation (CA) is an effective treatment for atrial fibrillation (AF) that traditionally requires fluoroscopic imaging to guide catheter movement and positioning. However, advances in electroanatomic mapping (EAM) technology and intracardiac echocardiography (ICE) have reduced procedural reliance on fluoroscopy. We conducted a prospective registry study of 162 patients enrolled at five centers proficient in high-volume, minimal-fluoroscopy CA between March 2016 and March 2018 for the CA of symptomatic, drug-refractory paroxysmal, or persistent AF that sought to assess the safety and efficacy of minimal- versus zero-fluoroscopy AF CA. We evaluated procedural details, acute procedural outcomes and complications, and one-year follow-up data. All operators used an EAM system (CARTO^®^; Biosense Webster, Irvine, CA, USA) and ICE. Ultimately, two patients did not pursue CA postenrollment. A total of 104 (66%) patients had paroxysmal AF with a mean ejection fraction of 58% ± 9%. Twenty-six (16.3%) patients were scheduled for repeat ablation. A total of 100 (63%) procedures were performed with zero fluoroscopy. The mean fluoroscopy time in the minimal-fluoroscopy group was 1.7 minutes ± 2.8 minutes. Further, the mean procedure duration was 192 minutes ± 37 minutes in the zero-fluoroscopy group and 201 minutes ± 29 minutes in the minimal-fluoroscopy group (p = 0.96). Pulmonary vein isolation was achieved in 153 patients (100%), with an acute procedural complication rate of 1.8%. One-year follow-up data were available for 152 (95%) patients with a mean follow-up time of 11.3 months ± 1.8 months. A total of 118 (76%) patients remained free from arrhythmia for up to 12 months, with no difference between the minimal- and zero-fluoroscopy cohorts (p = 0.18).

## Introduction

Catheter ablation (CA) for atrial fibrillation (AF) is an effective approach for the treatment of symptomatic, drug-refractory AF. The mainstay of effective therapy remains durable isolation of the pulmonary veins (PVs).^1^ Across a range of studies, freedom from AF following one or more PV ablation procedures ranges from 31% to 70% after one year.^2–4^

Catheter-based electrophysiology (EP) procedures have typically relied upon fluoroscopic imaging to guide catheter movement and positioning. Reported fluoroscopy times per case can be greater than 60 minutes, corresponding to skin radiation doses of 1,000 mGy ± 500mGy in the right anterior oblique and 1,500mGy ± 400mGy in the left anterior oblique projections, respectively, for AF ablation procedures.^5,6^ Obese patients may be exposed to higher levels of radiation due to a markedly increased dose area product. Recently, several technologic advancements have enabled greater reliance on electroanatomic mapping (EAM) to guide catheter movement.^7,8^ As a result, fluoroscopy usage has more recently diminished over time from more than 60 minutes to less than 20 minutes on average.^9^ The adoption of current technologies as well as the reinforcement of “as low as reasonably achievable” (ALARA) radiation usage principles in the catheterization laboratory has provoked a drive to minimize fluoroscopy use.

The risk of harmful effects to the patient from radiation exposure encompasses both deterministic and stochastic effects. Deterministic effects such as radiation skin injury, hair loss, or cataracts occur once a radiation threshold is exceeded. Many case reports have noted the occurrence of radiation skin injury resulting from high-exposure fluoroscopy.^6,10^ The development of cancer is a stochastic effect that is not associated with a particular threshold but which increases proportionately with the dose. In addition to patient risks, there are both operator and EP laboratory staff risks, including thyroid disorders, malignancy, cataracts, and others.^11^

The use of lead aprons exacts an orthopedic toll as well. Orthopedic-related complaints, time off from work, and the rate and extent of injuries are increased in catheterization laboratory staff as compared with other staff.^11^ Devices are available to reduce the orthopedic burden associated with lead aprons, including a “phone booth” lead box,^12^ a suspended lead apron system, or remote navigation systems.^13,14^ However, neither of the first two systems reduces the degree of radiation exposure experienced by personnel in the EP laboratory and none of these options reduces the level of radiation exposure to the patient.

Therefore, further reduction or even total elimination of fluoroscopy usage without compromising the efficacy or safety should be the goal. Multiple observational reports from single centers have demonstrated that very-low- or zero-fluoroscopy CA procedures are safe and acutely effective.^7,8,15–17^ To date, no systematic, multicenter, prospective study evaluating the safety and efficacy of a minimal- (or zero-) fluoroscopy–based approach to CA for AF has been performed. In this study, we postulated that radiofrequency CA for AF can be completed safely and effectively by multiple operators and institutions using low-/zero-fluoroscopy techniques. We predicted that, when using a technique that relies primarily upon intracardiac echocardiography (ICE) and EAM, fewer complications are likely to occur due to the increased safety of tracking systems that are superior to traditional fluoroscopy. We also aimed to compare outcomes among patients in our cohort whose procedures used minimal versus zero fluoroscopy.

## Methods

### Study design, patient, and site enrollment

This study is a clinical registry, with prospective enrollment of a single arm of consecutive patients between March 2016 and March 2018. Five high-volume AF ablation centers (> 100 cases per year) with physicians proficient in low-fluoroscopy AF ablation were identified. Specific techniques and technologies used were at the operators’ discretion, save for common use of an EAM system (CARTO^®^; Biosense Webster, Diamond Bar, CA, USA) and ICE. Patients (18–75 years) referred to undergo CA of symptomatic, drug-refractory paroxysmal or persistent AF who met guideline recommendations for CA of AF were identified and enrolled. The inclusion and exclusion criteria are listed in **Table 1**. Informed consent was obtained for all patients and the study was approved by each site’s local institutional review board. Further, the study protocol adhered to international guidelines on the study of human subjects. The target fluoroscopy exposure in the minimal-fluoroscopy arm was less than two minutes. If, due to procedural complexity, the two-minute fluoroscopy limit was exceeded, these patients were still considered for inclusion in the minimal-fluoroscopy arm according to the principle of ALARA.

### Ablation procedure

After obtaining informed consent, the selected patients were brought to the EP laboratory in a postabsorptive state and placed under general anesthesia. Esophageal monitoring was achieved using a variety of methods including a single-probe temperature monitor attached to a quadripolar catheter with a 1-cm offset from the distal tip of the catheter for visualization via EAM. This help to facilitate tracking and positioning of a multielectrode temperature probe (Circa, Englewood, CO, USA) along the esophagus in proximity to the ablation target or with ICE imaging of the esophagus. A number of common procedural approaches were adopted as part of this research. Vascular access via the femoral venous system was obtained using percutaneous ultrasound visualization. An ICE catheter (ACUSON AcuNav from Johnson & Johnson, New Brunswick, NJ, USA or CartoSound™ from Biosense Webster, Diamond Bar, CA, USA) was then advanced into the right atrium (RA) using ICE guidance, tactile feedback, and EAM guidance and the interatrial septum and left atrial (LA) structures were visualized on ICE. Next, a mapping and ablation catheter (Thermocool Smarttouch Surround Flow; Biosense Webster) was advanced into the RA, where a detailed EAM was created to showcase the RA and coronary sinus (CS) anatomy. Subsequently, a multipolar catheter was advanced into the CS using EAM and/or ICE guidance. Patients were either maintained on preprocedure oral anticoagulation or OAC therapy was disrupted within one to two days prior to the procedure and restarted immediately after the procedure. Intravenous heparin was given via bolus and continuous infusion to maintain an activated clotting time of greater than 350 seconds.

Transseptal puncture, either single or double, was then performed under ICE and EAM guidance. One of two transseptal puncture techniques was chosen as described previously.^18^ The more common technique selected is as follows, with all steps performed under ICE guidance: a standard 0.32-in guidewire (from either Abbott Laboratories, Chicago, IL, USA; Baylis Medical, Montreal, Canada; or Biosense Webster) was advanced into the RA and, once identified, directed into the superior vena cava (SVC). Once the wire was clearly identified in the SVC, a long sheath and dilator (8.5-Fr fixed-curve from Abbott Laboratories, Baylis, or Biosense Webster or Agilis from Abbott Laboratories) were advanced over the wire into the SVC. Then, after the wire was removed, a transseptal puncture needle (Brockenbrough; Abbott Laboratories or RF needle; Baylis) was advanced to just within the dilator tip. The unit was then “dragged” inferiorly and directed to the desired region of the interatrial septum. Once “tenting” was noted, puncture was performed, with confirmation of LA positioning via a subset of ICE visualization of dilator/sheath location, microbubble injection, wire advancement into a left pulmonary vein, or LA pressure waveform via a pressure transducer. Once the sheath was confirmed in the LA, the apparatus was flushed and connected to an air-free saline infusion line. If indicated, a second transseptal puncture was completed in a similar fashion.

As a less common technique for puncture, a long introducer sheath was introduced into the venous system over a long guidewire, then flushed and infused with air-free saline. The ablation catheter was then introduced through the long sheath into the RA and, under ICE and EA mapping guidance, the tip was directed to the interatrial septum. Once tenting of the intra-atrial septum was visualized by ICE, the sheath was advanced over the ablation catheter until it opposed the septum and covered the catheter. The ablation catheter was then removed and the dilator/needle, as above, was advanced into the interatrial septum, with subsequent puncture performed, as described above. This technique was most often used in the presence of pacing leads.

Following the placement of mapping and ablation catheters in the LA, a detailed EAM of the LA was created. Ablation was then performed primarily via a wide antral circumferential ablation (WACA) approach. Electrical isolation was established in all PVs via pacing maneuvers to confirm both entrance and exit block as well as pharmacologic testing via either adenosine or isoproterenol infusion. A 30-minute waiting period was required before confirming electrical isolation and “touch-up” ablation was performed as needed to achieve PV electrical isolation. Additional EP testing and ablation were completed at the discretion of the operator. Procedural parameters, including acute PV isolation success, ablation time, fluoroscopy time and dose, and complications, were all tracked. Ablation was done with contact-force-sensing catheters with mapping catheters, the type of which was left to the discretion of the operator.

### Tracking of procedural and clinical outcomes

After the procedure, patients were maintained on oral anticoagulation per guideline recommendations and every attempt was made to discontinue antiarrhythmic therapy postprocedure. If recurrences of atrial arrhythmias were observed after the index ablation procedure, additional ablation procedures, cardioversions, or the escalation of antiarrhythmic drug therapy were attempted at the discretion of the treating physician. Monitoring for AF recurrence was mandated at three to six months and at 12 months with ambulatory monitors per the operator’s discretion. Finally, additional clinically indicated electrocardiogram, ambulatory monitoring, or device interrogation data were recorded and periprocedural and follow-up complications were tracked.

In patients with existing pacing and/or defibrillator leads, prestudy fluoroscopic imaging and device interrogation were conducted as baseline measurements per the study protocol. During the transseptal puncture, lead visualization was performed using ICE if possible. At the conclusion of the procedure, fluoroscopic imaging and device interrogation were performed again (necessitating a small dose of radiation in this subset of patients).

### Statistical analysis

Baseline and procedural characteristics of patients were summarized as mean ± standard deviation for continuous variables, with highly skewed variables expressed as medians with the first and third quartiles (25%–75%). Categorical variables were expressed as frequencies/percentages. All data were collected in REDCap version 8 (REDCap, Nashville, TN, USA) in a de-identified fashion. All analyses were performed using the Statistical Package for the Social Sciences version 18.0 for Windows (IBM Corp., Armonk, NY, USA).

## Results

A total of 167 patients were screened for participation, with 162 patients across five institutions consenting to enrollment, and six primary operators were included. Two patients subsequently did not want to pursue CA after study enrollment (n = 160 treated). Three sites were academic teaching centers, with fellows participating in the procedures, and two sites were private institutions without trainees. Patient demographics are shown in **Table 2**. Of the 162 patients enrolled, data were available for 160 patients. Complete follow-up assessments through one year were performed for 152 (93%) patients. The mean patient age was 62.1 years ± 8.6 years, with 62.5% being male; further, the study population presented a mean CHA_2_DS_2_-VASc score of 1.9 ± 1.3 points, a mean LVEF value of 58% ± 9%, and a mean LA dimension of 4.4 cm ± 0.8 cm. Persistent AF accounted for the primary concern in 34.4% of the cases. There were 26 patients (16.3%) who had undergone previous AF ablations and three (1.9%) patients had existing intracardiac devices (either a pacemaker or implantable cardioverter-defibrillator).

Our protocol design enabled operators to use their preferred tactics and tools to achieve minimal fluoroscopy such that a variety of approaches were adopted across the different included cases as described in the Methods. In **Table 3**, the key procedural details to achieving minimal-fluoroscopy AF ablation are listed. EAM and ICE were used by all study sites. All study sites also used some low- or zero-fluoroscopic approach to monitor either esophageal position or temperature. Contact-force ablation RF catheters were used by all sites. Double transseptal puncture was performed in 45.6% of cases. In addition, almost all sites performed a WACA approach and all sites completed both electrical pacing and pharmacologic testing to confirm PV isolation.

Intraprocedural outcomes, including procedural time, RF time, and fluoroscopy exposure (reported as both fluoroscopy time and total radiation dose), are shown in **Table 4**. The mean procedure duration was 192 minutes ± 37 minutes in the zero-fluoroscopy arm and 201 minutes ± 29 minutes in the minimal-fluoroscopy arm (p = 0.96). Acute PV isolation was achieved in 100% of the cases, with the PV isolation status being unclear during case reporting in seven patients. WACA lines were completed in 96% of the cases, whereas segmental ablation was completed in 4% of cases. Additional ablation lesions included cavotricuspid isthmus ablation (23%), LA roof line (19%), LA septal line (4%), and LA mitral isthmus line (0.2%). Of the 160 procedures, 100 (63%) were performed with zero fluoroscopy. In 50% of the procedures performed, the operator and EP laboratory staff removed lead aprons prior to creating the first ablation lesion.

**Figure 1:**
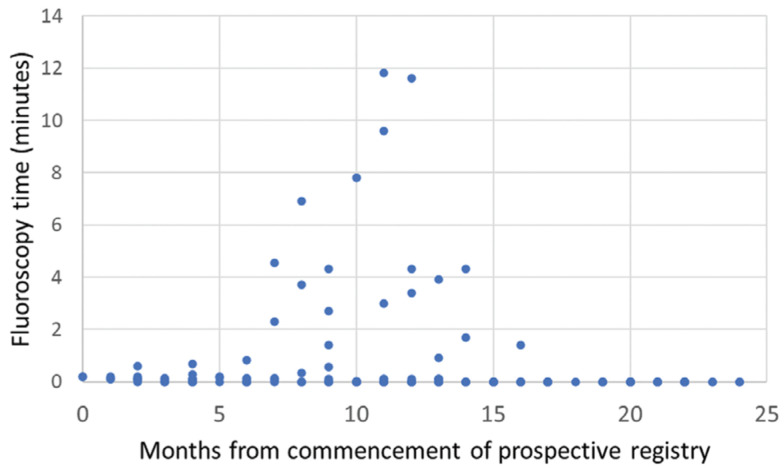
Fluoroscopy times in the patients enrolled for AF ablation in the first year of the prospective registry (median: 0.1 minutes, interquartile range: 0.2 minutes, mean: 0.9 minutes) were higher than those for patients enrolled in the second year of the registry (median: 0.0 minutes, interquartile range: 0.0 minutes, mean: 0.4 minutes; p = 0.01).

**Figure 2:**
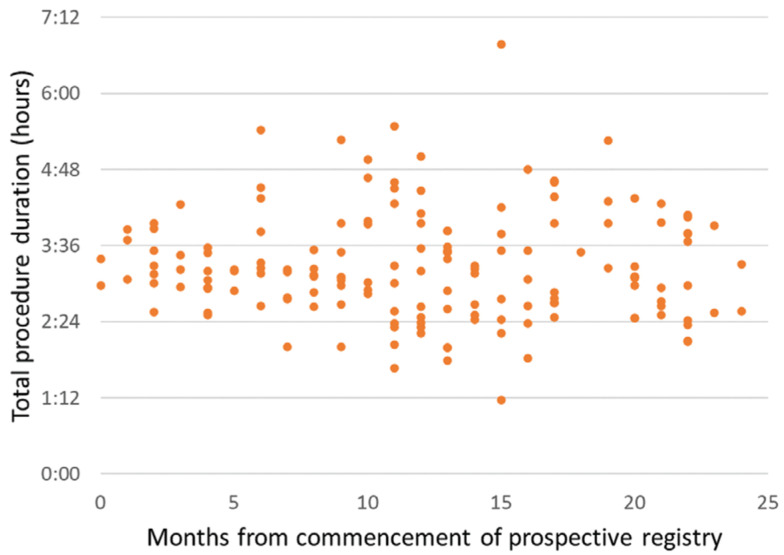
The total procedure times for patients enrolled for AF ablation in the first year of the prospective registry (3.19 ± 0.3 hours) were similar to those for patients enrolled in the second year of the registry (3.13 ± 0.04 hours; p = 0.6).

**Figure 3:**
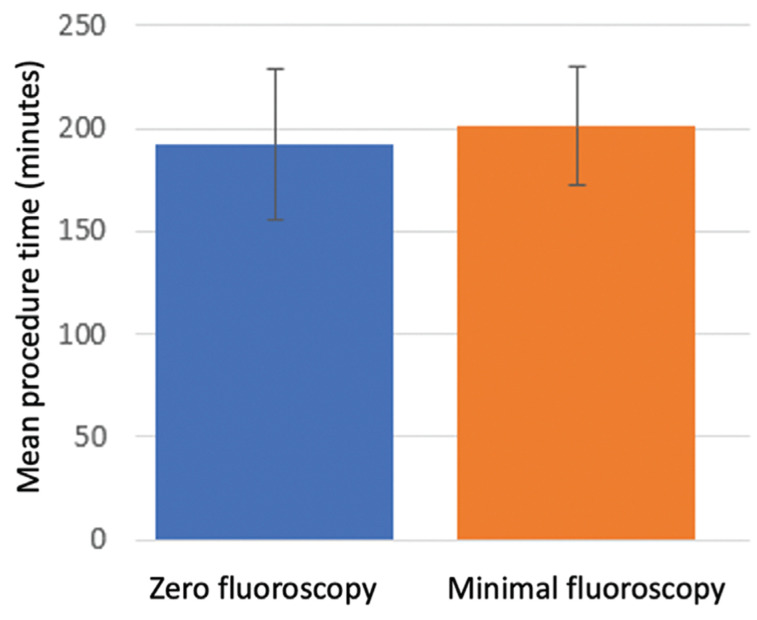
The mean procedure times for the minimal- and zero-fluoroscopy cohorts were similar (192 ± 37 minutes versus 201 ± 29 minutes; p = 0.96).

**Figures 1** and **2** show the fluoroscopy time and procedure time of each patient as a function of time (months of enrollment in the study) after the commencement of the registry across all centers. Fluoroscopy times were significantly shorter for patients enrolled in the second half of the study as compared with those included during the first half (median: 0 minutes, interquartile range: 0 minutes versus median: 0.1 minutes, interquartile range: 0.2 minutes; p = 0.01) **(Figure 1)**. The total procedure duration did not significantly change over the course of the first and second years of the study **(Figure 2)**, despite a significant reduction in fluoroscopy time. When PV isolation and procedural complication rates were assessed as a function of the duration of fluoroscopy exposure, no statistical differences in outcomes were noted between the zero- and minimal-fluoroscopy cohorts. **Figure 3** shows the mean procedure time as a function of fluoroscopy exposure, revealing that no statistically significant difference existed regarding procedure time between the two groups. Implanted cardiac rhythm management devices were present in three (2%) patients, with no device lead parameter changes or dislodgements occurring postablation.

Univariate correlates for any fluoroscopy use included the conduct of double transseptal puncture (versus single transseptal puncture; p < 0.01), an earlier index procedure date from the initiation of the registry (p < 0.01), the absence of a trainee (p = 0.03), and lower patient body weight (p = 0.01). During multivariate analysis, the conduct of double transseptal puncture (p < 0.01) and an earlier index procedure date from the initiation of the registry (p < 0.01) remained independent correlates of any fluoroscopy use. The presence of a trainee during the operation was not associated with increased fluoroscopy use. On the contrary, centers that involved trainees in the procedures had lower total durations of fluoroscopy use relative to those centers with no trainees involved (Spearman’s rho = −0.16; p = 0.04).

**Figure 4:**
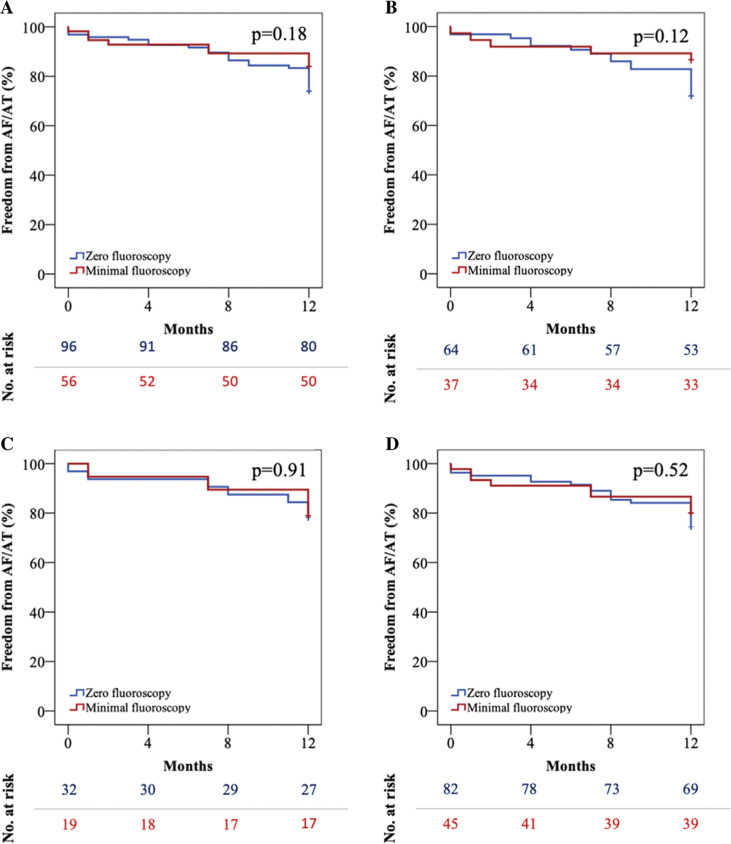
Kaplan–Meier survival curves showing freedom from atrial arrhythmias in **A:** all patients in the registry **B:** patients with paroxysmal AF**, C:** patients with persistent AF, and **D:** patients with no prior ablation stratified by zero- versus minimal-fluoroscopy use. There was no significant difference between zero- and minimal-fluoroscopy use among groups by log-rank test.

Clinical outcomes, as predefined by the study design, are shown in **Table 4**. Acute procedural complications occurred in three patients (1.8%), including one case of transient ischemic attack, one case of groin hematoma, and one case of pericardial effusion without hemodynamic effect, all of which were managed conservatively. Follow-up data from up to one year postprocedure were available from 152 patients; 76% of patients remained free from AF based on symptom correlation and cardiac monitoring at three to six months and at 12 months (95% confidence interval: 68%–82%). At 12 months of follow-up, 93% of patients were in sinus rhythm, while 59% of patients were on antiarrhythmic medications. As shown in **Figure 4**, the rate of AF recurrence at one year of follow-up was not significantly different between the zero-fluoroscopy group and the fluoroscopy-exposed group (p = 0.18, log-rank test). This remained true within the subcohorts of paroxysmal AF (p = 0.12) and persistent AF (p = 0.91) and among patients without a history of prior ablation (p = 0.52). Over the course of one year, 14 patients (9%) were hospitalized for arrhythmia-related issues, including antiarrhythmic medication initiation/escalation. Additionally, 16 patients (10%) required cardioversion and seven patients (4%) underwent repeat ablation procedures. There was one procedurally unrelated death that occurred due to cardiac arrest at six months after the index ablation in a patient with known coronary artery disease who was off rhythm-control medications. The late complication rate at one year was 2.6%, with one case of transient ischemic attack, one case of myocardial infarction and congestive heart failure exacerbation, and one episode of pericarditis.

## Discussion

To our knowledge, this is the first systematic, multicenter, prospective evaluation of a minimal-/zero-fluoroscopy approach to RF CA of AF reporting long-term clinical outcomes. Both academic and private centers were included and all operators who participated were experienced in adopting the minimal-/zero-fluoroscopy approach to CA. A total of 162 patients were enrolled, with one-year clinical follow-up data collected after the index ablation, including concerning predetermined clinical follow-up and ambulatory monitoring. Previous reports of nonfluoroscopic catheter visualization involved prerecorded X-ray imaging using the MediGuide system (Abbott Laboratories, Chicago, IL, USA)^16^ or were single-center, single-operator studies lacking long-term follow-up data.^19,20^

We speculate that procedural outcomes in our registry suggest equivalency to historically reported conventional CA with regard to PV isolation, overall procedure times, and ablation times, including when considering ablation for both paroxysmal and persistent AF,^3,4,21^ which can pave the path for future large randomized controlled studies. Fluoroscopy use was significantly lower in this investigation than among historical controls, with further reductions seen during the course of the enrollment period. This suggests a further reduction and/or elimination of fluoroscopy use with experience, while simultaneously maintaining safety and efficacy, essentially constituting progression on a continuous learning curve. The overall procedure times were also comparable to historically reported times. Notably, 63% of procedures were performed completely without fluoroscopy, with operators and staff able to avoid using lead aprons, whereas 90% of procedures required between zero and two minutes of fluoroscopy such that majority of the procedures were performed with either zero or very low fluoroscopy (< 2 minutes), with just one patient requiring as much as 11.6 minutes of fluoroscopy. When fluoroscopy was used, in 98% of these cases, it was adopted entirely prior to the first ablation lesion in the LA but not necessarily prior to accessing the LA. For example, in some of these cases, minimal fluoroscopy following transseptal puncture was used to mark the esophageal shadow on the LA EAM, a step which follows transseptal puncture but precedes the first LA ablation lesion.

Long-term clinical outcomes were also equivalent to those of historical controls as measured by freedom from AF at up to 12 months using standard, accepted means of follow-up.^3,4,21^ To our knowledge, this study is the first report of such long-term clinical outcomes after minimal-/zero-fluoroscopy AF ablation, demonstrating no compromise in long-term outcomes.

Complication rates were significantly lower than historically reported rates for conventional ablation.^22,23^ We hypothesize that this may stem from a better understanding of catheter–tissue relationships achieved through increased reliance on ICE and EAM as well as greater use of contact-force ablation catheters. We further posit that reliance on fluoroscopy for catheter manipulation may impart a false sense of security in that tissue borders are not readily visualized, potentially contributing to heightened risks of perforation and collateral tissue injury. With the integrated use of ICE, EAM, and contact-force-sensing technologies, the ability to visualize and quantify catheter–tissue interactions in real time is more accurate and reliable than when using fluoroscopic imaging. This study was not designed to assess reductions in the short-term or long-term consequences of radiation exposure or lead apron use among patients or EP laboratory staff.

This study was intentionally designed as a single-arm, prospective study, as the authors felt that introducing a control arm of patients undergoing conventional fluoroscopy-based ablation would be challenging. The authors considered enrolling a control group for conventional fluoroscopic-guided AF ablation but felt that reverting to a fluoroscopy-based approach performed by the same providers to keep the procedures otherwise uniform would be exposing patients to radiation only for the purpose of the randomization, without actual meaningful use of fluoroscopy. Also, a strategy involving “conventional” operators as controls was believed to potentially introduce confounders of operator procedural variability and, hence, risk difficult-to-interpret findings. To provide perspective, we compared the outcomes of zero- and minimal-fluoroscopy patient populations within our study group and there were no significant differences in clinical outcomes between these populations. Moreover, the overall clinical outcomes were comparable to previously reported outcomes for conventional ablation, with lower complication rates. Our data suggest that the additional step of complete elimination of fluoroscopy beyond “very low” use did not compromise safety or efficacy.

All operators in this study were experienced with minimal-/zero-fluoroscopy approaches at the outset of the study, with levels of experience ranging from one to five or more years performing 100 to 200 procedures a year using zero or minimal fluoroscopy. The authors understand that a learning curve exists for the adoption of these approaches. Of note, further fluoroscopy reduction was evident even among these experienced operators during the course of this study. Although not systematically evaluated, the authors all clearly honed their techniques in a fashion deemed safe while maintaining efficacy during their respective learning curves. It would be reasonable to presume that, for most operators interested in adopting a low-fluoroscopy approach, similar outcomes are likely to be achieved. Further study and confirmation are warranted. Moreover, the evaluation of minimal-/zero-fluoroscopy approaches to the ablation of other cardiac arrhythmias is important.

Minimal-/zero-fluoroscopy ablation can be accomplished using other existing mapping systems as has been demonstrated in the literature.^7,8,24^ Extrapolation of these findings to the ablation of other cardiac arrhythmias or other ablation technologies (eg, cryoablation) is not possible at this time; however, it is reasonable to assert that the core techniques used here likely can be transferred to other ablation procedures. Indeed, case series describing minimal-fluoroscopy ablation of supraventricular tachycardia, atrial flutter, and ventricular tachycardia exist in the literature already.^25,26^ During the study enrollment period, further advancements in CA technology and techniques have been proposed and evaluated, including high-power, short-duration ablation; the use of various balloon catheters; and the involvement of electroporation technology.^1,27–29^ A minimal-fluoroscopy approach using these technologies has not yet been evaluated systematically; however, given the capability to leverage EAM and ICE with these technologies, these technologies should permit the addition of a minimal-fluoroscopy approach as well.

As more evidence points to the safety and efficacy of a minimal-/zero-fluoroscopy approach to CA, it becomes increasingly difficult to argue against widespread acceptance and adoption, with a minimal- or zero-fluoroscopy approach becoming standard of practice. In this study, this approach when used specifically for AF ablation has been shown to be safe and effective, without compromising procedural efficiency, across both academic and private settings and with variations in technique.

### Limitations

This study reports by far the largest cohort of patients undergoing minimal-/zero-fluoroscopy ablation for AF but, with only 162 enrolled patients, this investigation still involves a relatively small population undergoing a frequently performed procedure. Further validation across additional patients, centers, and operators is indicated. A heterogeneous population of patients with both paroxysmal and persistent AF as well as patients undergoing initial and repeat procedures was included, potentially diluting the overall findings. However, the authors felt that the inclusion of a spectrum of common scenarios encountered in a high-volume AF ablation center would be more reflective of typical practice patterns and, hence, render the findings more generalizable. The cohort also includes a balanced mix of patients from both academic-teaching and community hospitals and, therefore, better represents real-world AF ablation procedures.

A single-arm design was chosen for this study, with the rationale as described. The typical limitations of such a study design are to be expected here, including nonexperimental design, difficulty controlling for confounding factors in a heterogenous patient cohort, selection bias, and underreporting as nonregistered patients can often have worse outcomes.

Reliance on EAM, ICE, and contact force were critical to achieve safe and efficacious results. It may be argued that this approach, particularly with respect to ICE, may add costs to the procedure. We note that, at least in the United States, EAM systems and ICE have become standard of care for AF ablation and, therefore, no significant added costs are incurred. Moreover, in this study, the CARTO^®^ mapping system (Biosense Webster, Diamond Bar, CA, USA) was used exclusively over other mapping systems. The authors, however, do not intend to suggest the superiority of one system over others. Several other reports—primarily single-center, retrospective cohort studies—have used alternative mapping systems such as NavX™ EnSite™ (Abbott Laboratories, Chicago, IL, USA) and the Rhythmia™ mapping system (Boston Scientific Corp., Natick, MA, USA).^8,30,31^ Radiofrequency with point-by-point ablation was the only ablative energy modality used in this study, although successful PVI with cryoballoon and laser balloon technologies has been reported in conjunction with zero- or minimal-fluoroscopy use in recent series.^31,32^

The detailed timing of each portion of the procedure, such as the LA dwell time, was not tracked in this registry, which could differ between cohorts subjected to zero versus minimal fluoroscopy. Also, patients with implantable cardiac devices made up only a small percentage (1.9%) of our cohort and the safety of zero-fluoroscopy ablation would need to be further assessed in a larger cohort of these individuals. Finally, the reported PVI isolation rates in our dataset may not reflect the true rates, as seven patients had incomplete intraprocedural data reported.

## Conclusions

We present the first prospective, multicenter study evaluating the safety and efficacy of a minimal-/zero-fluoroscopy approach to RF CA for AF. This approach was found to be both safe and effective, with the ability to significantly reduce or eliminate the use of fluoroscopy during these procedures. Moreover, acute procedural complications were lower than those of historical controls and procedural efficiency was not compromised. The further evaluation of this approach for managing AF and in other ablation procedures should also be explored. Given the continued advancement in technology that is ongoing, it is our sincere hope that our field will progress toward a point at which the use of fluoroscopy will be considered the exception rather than the rule. Given the known and unknown risks of radiation exposure to patients, EP laboratory staff, and physicians, the authors believe that pursuing a low-fluoroscopy approach is the appropriate direction for the field of cardiac EP.

## Figures and Tables

**Table 1:** Study Inclusion and Exclusion Criteria

Inclusion Criteria	
•	Patient meets accepted AHA/ACC/HRS guideline indications for catheter ablation of paroxysmal or persistent AF
•	First or subsequent AF ablation procedure
•	Aged 18–75 years
•	Patient has the capacity to provide informed consent
**Exclusion Criteria**	
•	Unable to provide informed consent
•	History of mechanical mitral valve replacement (unable to rely on EAM only)
•	Documented left atrial thrombus
•	Prior ASD repair
•	Known venous malformations or implanted instrumentation (IVC filter)
•	Known PV stenosis
•	Pregnancy
•	NYHA functional class ≥ 3
•	CHF within 90 days, MI within the past 90 days, or coronary revascularization within 90 days
•	Patients who are undergoing cryoablation for AF or non–standard-of-care ablation (eg, FIRM ablation)

Note: History of implanted pacemaker or ICD with transvenous leads was not an exclusion criterion but we did track patients treated with preexisting transvenous leads with the hypothesis that, although fluoroscopy use will be higher in these patients than in those without leads, it will still be lower than in conventionally treated patients.

AHA/ACC/HRS: American Heart Association/American College of Cardiology/Heart Rhythm Society; AF: atrial fibrillation; ASD: atrial septal defect; CHF: congestive heart failure; EAM: electroanatomic mapping; FIRM: focal impulse and rotor modulation; ICD: implantable cardioverter-defibrillator; IVC: inferior vena cava; MI: myocardial infarction; NYHA: New York Heart Association; PV: pulmonary vein.

**Table 2:** Baseline Characteristics

	Zero Fluoroscopy (n = 100)	Minimal Fluoroscopy (n = 60)	p-value
Age (mean ± SD)	62 ± 8.4 years	63 ± 8.9 years	0.3
Male sex, n (%)	65 (65)	36 (60)	0.4
Paroxysmal AF	66 (66)	38 (63)	0.4
Baseline medications, n (%)			
Flecainide	21 (21)	5 (8)	< 0.05
Sotalol	16 (16)	3 (5)	< 0.05
Amiodarone	9 (9)	5 (8)	0.3
Metoprolol	42 (42)	23 (38)	0.5
Diltiazem	7 (7)	8 (13)	0.3
Preprocedure anticoagulation or antiplatelet use, n (%)			
Apixaban	39 (39)	17 (28)	0.2
Rivaroxaban	33 (33)	19 (32)	0.5
Dabigatran	10 (10)	5 (8)	0.6
Warfarin	12 (12)	8 (13)	0.7
Aspirin	0 (0)	1 (2)	0.5
None	2 (2)	6 (10)	0.2
Comorbidities, n (%)			
Coronary artery disease	22 (22)	8 (13)	0.1
Myocardial infarction/PCI/PTCA	11 (11)	4 (7)	0.5
CABG	3 (3)	2 (3)	0.5
Hypertension	72 (72)	25 (42)	0.1
Congestive heart failure	3 (3)	3 (5)	0.5
Diabetes mellitus	23 (23)	10 (17)	0.2
Stroke	3 (3)	2 (3)	0.5
Previous cardioversion for AF	46 (46)	21 (35)	0.2
Previous AF ablation	16 (16)	10 (17)	0.5
Pacemaker or ICD in situ	1 (1)	2 (3)	0.5
LVEF (mean ± SD)	58% ± 9%	57% ± 7%	0.2
LVEF, n (%)			
; 55%, n (%)	76 (76)	54 (90)	
40%–55%, n (%)	15 (15)	3 (5)	
30%–40%, n (%)	5 (5)	3 (5)	
< 30%, n (%)	2 (2)	0	
CHA_2_DS_2_-VASc score (mean ± SD)	1.9 ± 1.3	1.7 ± 1.5	0.5
CHA_2_DS_2_-VASc score, n (%)			
0	15 (15)	11 (18)	
1	33 (33)	23 (38)	
2	13 (13)	10 (17)	
3	28 (28)	10 (17)	
4	7 (7)	4 (7)	
5	2 (2)	2 (3)	
6	2 (2)	0	
LA volume index (mean ± SD)	39 ± 12 mL/m^2^	37 ±10 mL/m^2^	0.5
LA dimension (mean ± SD)	4.4 ± 0.9 cm	4.2 ± 0.7 cm	0.3

AF: atrial fibrillation; CABG: coronary artery bypass graft; ICD: implantable cardioverter defibrillator; LA: left atrium; LVEF: left ventricular ejection fraction; PCI: percutaneous coronary intervention; PTCA: percutaneous transluminal coronary angioplasty; SD: standard deviation.

**Table 3:** Procedural Parameters

	Zero Fluoroscopy (n = 100)	Minimal Fluoroscopy (n = 60)	p-value
Conscious sedation, n (%)	22 (22)	3 (5)	< 0.05
General anesthesia, n (%)	78 (78)	57 (95)	0.5
Vascular access with fluoroscopic landmarks, n (%)	0 (0)	5 (8)	0.1
ICE used for transseptal puncture, n (%)	100 (100)	56 (93)	0.4
Patients with preexisting PPM/ICD, n (%)	1 (1)	2 (3)	0.5
Double transseptal, n (%)	17 (17)	55 (92)	0.2
Presenting rhythm sinus, n (%)	63 (63)	36 (60)	0.4
Esophageal monitoring, n (%)			
CartoSound™	55 (55)	15 (25)	0.1
Multielectrode catheter	23 (23)	37 (62)	0.2
EAM	9 (9)	1 (2)	< 0.05
Other (Esophastar™ catheter, single electrode)	12 (12)	7 (12)	0.5
Preprocedure imaging, n (%)			
TEE	44 (44)	38 (63)	0.7
CT	17 (17)	50 (83)	0.1
MRI	21 (21)	0 (0)	< 0.05

CT: computed tomography; EAM: electroanatomical mapping; ICD: implantable cardioverter defibrillator; ICE: intracardiac echocardiography; MRI: magnetic resonance imaging; PPM: pacemaker; TEE: transesophageal echocardiogram.

CartoSound is a product of Biosense Webster (Diamond Bar, CA, USA). Esophastar is a product of Johnson & Johnson (New Brunswick, NJ, USA).

**Table 4:** Clinical Outcomes

	Zero Fluoroscopy (n = 100)	Minimal Fluoroscopy (n = 60)	p-value
Procedure time (mean ± SD)	192 ± 37 min	201 ± 29 min	0.96
Fluoroscopy time (mean ± SD)	0 min	1.7 ± 2.8 min	< 0.05^**^
Median radiation dose (median, IQR)	0 mGy · cm^2^	246.55 (79–564) mGy · cm^2^	< 0.05^**^
Ablation lesions (data available for n = 153 patients), n (%)			
LSPV isolated^*^	96 (100)	57 (100)	0.5
LIPV isolated^*^	96 (100)	57 (100)	0.5
RSPV isolated^*^	96 (100)	57 (100)	0.5
RIPV isolated^*^	96 (100)	57 (100)	0.5
CTI ablated	20 (21)	18 (31)	0.06
LA roof line	21 (22)	9 (16)	0.2
Mitral isthmus line	3 (3)	0 (0)	0.2
LA septal line	4 (4)	4 (7)	< 0.05
WACA	94 (98)	55 (97)	0.4
Segmental ablation	3 (3)	3 (5)	0.3
Fluoroscopy usage			
Operator and EP laboratory staff removed lead aprons prior to first ablation, n (%)	N/A	58 (98)	< 0.05^**^
Acute procedural complications			
Pericardial effusion	0	1	0.4
Transient ischemic attack	1	0	1.0
Groin hematoma	0	1	0.4
Follow-up, n (%)			
One-year follow-up data available	95 (95)	57 (95)	
Freedom from all atrial arrhythmias during follow-up	71 (75)	44 (77)	0.5
Sinus rhythm at 12 months	90 (95)	51 (89)	0.4
Recurrent AF during follow-up requiring cardioversion (%)	16 (17)	0	< 0.05
Repeat AF ablation (%)	5 (5)	2 (4)	0.3
Late complications at one year			
Transient ischemic attack	1	0	1.0
Myocardial infarction	0	1	0.4
Heart failure exacerbation	1	0	1.0
Pericarditis	0	1	0.4

AF: atrial fibrillation; CTI: cavotricuspid isthmus; EP: electrophysiology; IQR: interquartile range; LA: left atrium; LIPV: left inferior pulmonary vein; LSPV: left superior pulmonary vein; RIPV: right inferior pulmonary vein; RSPV: right superior pulmonary vein; SD: standard deviation; WACA: wide-area circumferential ablation.

^*^Reported PV isolation rates from sites with seven patients having unclear/undetermined PV isolation status.

^**^p-value is given for illustrative purposes only, as this test was confounded.
